# The effects of gestational diabetes mellitus with maternal age between 35 and 40 years on the metabolite profiles of plasma and urine

**DOI:** 10.1186/s12884-022-04416-5

**Published:** 2022-03-02

**Authors:** Xiao-ling He, Xiao-jing Hu, Bai-yu Luo, Yin-Yin Xia, Ting Zhang, Richard Saffery, Jamie De Seymour, Zhen Zou, Ge Xu, Xue Zhao, Hong-bo Qi, Ting-Li Han, Hua Zhang, Philip N. Baker

**Affiliations:** 1 Department of Obstetrics and Gynecology, The First Affiliated Hospital of Chongqing Medical University, No. 1 Youyi Road, Yuzhong District, Chongqing, 400016 People’s Republic of China; 2 State Key Laboratory of Maternal and Fetal Medicine of Chongqing Municipality, Chongqing Medical University, Chongqing, 400016 China; 3 School of Public Health and Management, Chongqing Medical University, Chongqing, 400016 China; 4 Cancer & Disease Epigenetics, Murdoch Children’s Research Institute and Department of Pediatrics, University of Melbourne, Melbourne, VIC Australia; 5 College of Health, Massey University, Wellington, New Zealand; 6 Institute of Life Sciences, Chongqing Medical University, Chongqing, 400016 China; 7 Department of Obstetrics and Gynecology, The Second Affiliated Hospital of Chongqing Medical University, Chongqing, China; 8 College of Life Sciences, University of Leicester, Leicester, UK

**Keywords:** Metabolomics, Gestational diabetes mellitus, Pregnancy, Advanced maternal age

## Abstract

**Background:**

Gestational diabetes mellitus (GDM) is defined as impaired glucose tolerance in pregnancy and without a history of diabetes mellitus. While there are limited metabolomic studies involving advanced maternal age in China, we aim to investigate the metabolomic profiling of plasma and urine in pregnancies complicated with GDM aged at 35–40 years at early and late gestation.

**Methods:**

Twenty normal and 20 GDM pregnant participants (≥ 35 years old) were enlisted from the Complex Lipids in Mothers and Babies (CLIMB) study. Maternal plasma and urine collected at the first and third trimester were detected using gas chromatography-mass spectrometry (GC-MS).

**Results:**

One hundred sixty-five metabolites and 192 metabolites were found in plasma and urine respectively. Urine metabolomic profiles were incapable to distinguish GDM from controls, in comparison, there were 14 and 39 significantly different plasma metabolites between the two groups in first and third trimester respectively. Especially, by integrating seven metabolites including cysteine, malonic acid, alanine, 11,14-eicosadienoic acid, stearic acid, arachidic acid, and 2-methyloctadecanoic acid using multivariant receiver operating characteristic models, we were capable of discriminating GDM from normal pregnancies with an area under curve of 0.928 at first trimester.

**Conclusion:**

This study explores metabolomic profiles between GDM and normal pregnancies at the age of 35–40 years longitudinally. Several compounds have the potential to be biomarkers to predict GDM with advanced maternal age. Moreover, the discordant metabolome profiles between the two groups could be useful to understand the etiology of GDM with advanced maternal age.

**Supplementary Information:**

The online version contains supplementary material available at 10.1186/s12884-022-04416-5.

## Introduction

With the change of birth policies that came into effect in January 2016, Chinese couples are now legally allowed to have two children. However, a direct legacy of previous policies is that many couples now aiming for a second child are aged in their late 30s or 40s. The result is an increasing number of pregnancies with advanced maternal age (AMA) , defined as maternal age greater than or equal to 35 years at the expected date of delivery [1]. The WHO estimated the global prevalence of AMA pregnancies at approximately 12.3% in 2014 [2]. A 12-year cohort study indicated the incidence of pregnancies with AMA rose from 6.5% to 17.2% in Southwestern China in 2019, surprisingly, among the pregnancies greater than or equal to 35 years, most of them are between 35-40 years, while merely a small proportion (10%) are over 40 years. This implies the characteristics of pregnancies between 35-40 years can represent the overall profiling of AMA [3]. It is well known that pregnancy complications and adverse pregnancy outcomes are closely correlated with advanced maternal age. A retrospective cohort study found that pregnancy complications such as preeclampsia and gestational diabetes mellitus (GDM) were at higher risk in pregnant women with AMA [4]. GDM, an abnormal glucose tolerance first recognized in pregnancy, has been found to be 2-3 fold higher in AMA group compared with those aged 20-30 years in China [5, 6]. Furthermore, there is an increasing recognition of the increased risk of obesity, glucose intolerance, and cardiovascular disorders among maternal and offspring of GDM with advanced maternal age [7, 8].

Metabolomics is an effective approach to investigate low molecular metabolites representing the metabolic status of cells, tissues, or organisms. Recently, it is gaining popularity as a screen tool to investigate metabolic changes and biomarker discovery for GDM. Blood is the most commonly used biospecimen in the GDM metabolomic studies due to the fact that it usually contains the highest concentration of various classes of metabolites. Zhao *et al* performed a metabolomics analysis of serum from 107 cases diagnosed with GDM compared with 107 healthy controls. They found that six amino acids and uric acid in the case group, were significantly lower than those in the control group [9]. Secondly, urine is another popular specimen for metabolomics research because of its non-invasive sampling and effortless preparation (a single dilution step) prior to mass spectrometry analysis. Sakurai K *et al* investigated urine metabolomic profiling from 121 GDM cases and 121 healthy controls in Japan. They proposed that 1,3-diphosphoglycerate and ethanolamine in urine were able to discriminate GDM from healthy controls [10]. Moreover, our group explored urine metabolomic profiling by comparing 27 GDM cases with 34 healthy controls. The results suggested that uric acid resulting from the catabolism of purine nucleosides could accurately classify GDM patients [11].

Despite considerable previous GDM metabolomic studies, there are few exclusively investigating the metabolic profiling of GDM in pregnancies of AMA. Furthermore, the ability to expose GDM in such pregnancies has not priorly been assessed. Here, we explored the metabolomic profiling of plasma and urine in pregnancies complicated with GDM aged at 35-40 years at early and late gestation using a GC-MS based metabolomics approach.

## Materials and methods

### Study participants

In this prospective case-control study, GDM (n=20) and healthy (n=20) pregnant women ≥ 35 years were recruited from Complex Lipids in Mothers and Babies (CLIMB) project (ChiCTR-IOR-16007700) [12]. Maternal age, body mass index (BMI), gestational age and blood pressure (BP) were matched. Fasted blood and urine specimens were collected at the first (11-14 gestational weeks) and third trimesters (32-34 gestational weeks). During 24-28 gestational weeks, a 75 g oral glucose tolerance test (OGTT) was conducted. GDM was diagnosed according to the International Association of Diabetic Pregnancy Study Group (IADPSG) guidelines (at least fasting blood glucose ≥ 5.1 mmol/L, or 60 minutes post 75 g OGTT blood glucose level ≥ 10 mmol/L, or 120 minutes post 75 g OGTT blood glucose level ≥ 8.5mmol/L). Multiple pregnancies or pregnancies with other complications were excluded.

### Sample preparations for plasma and urine

Aliquots (100 μL) of thawed urine were mixed with 80 μl 3M NaOH and 20 μL of the internal standard (IS, 2,3,3,3-d4-alanine,10 mM). The prepared liquid mixtures were stored at −80°C. Aliquots (100 μL) of thawed plasma were mixed with 20 μL of IS. To eliminate protein, 400 μL of cold methanol was added and tubes were placed at −20 °C for 30 minutes. A total of 350 μl of supernatant was isolated following centrifugation (4000 rpm for 20 mins). Quality controls (QC) were also prepared from sub-aliquoting 20 μL of each biofluid (urine or plasma) into the collection tubes. All QC samples were prepared as the identical procedure regarding different sample types. Lastly, plasma supernatant was concentrated by in a speedvac (LABCONCO) at a speed of 4000 rpm for 8 h and 3 h respectively. All extracted biological samples were stored at – 80 °C before derivatization.

Prior to GC-MS analysis, all samples were derivatized with MCF as published by Smart *et al* [13]. An Agilent GC7890B chromatograph coupled to a MSD5977A mass spectrometry with electron impact ion source set at 70 eV was used for the analysis of MCF derivatives [14].

### Statistical analysis

The GC-peaks were deconvoluted by Automated Mass Spectral Deconvolution and Identification System (AMDIS) and identified using our MCF mass spectra library (built by chemical standards) and commercial National Institute of Standards and Technology library (NIST14 library, https://www.nist.gov/nist-research-library). The metabolite identification was based on their MS spectrum > 90 % similarity and within a 30s retention time bin to its respective compound in the MS spectral library. After the removal of contamination from blank, metabolite concentration was first normalized by the level of IS, and the batch effect was minimized using median centering of QC samples [15]. Lastly, the dilution effect of urine and plasma was corrected by total ion count. The metabolite levels were first transformed into Gaussian distribution using log and Pareto scalings and the differences of metabolite abundance in GDM and healthy controls were calculated using Student’s T-test. To avoid false-positive results from multiple statistical tests, false discovery rates (FDR) for each metabolite were calculated by qvalue R package [16]. The area below the receiver operating characteristic (ROC) curve was calculated using the pROC R-package [17]. Metabolites were linked to their corresponding metabolic pathways using Kyoto Encyclopedia of Genes and Genomes (KEGG) databased. Line plot, heatmap, and chord plot were illustrated using ggplot2 R-based packages [18]. Moreover, the power analysis of 75g OGTT (120 min postprandial blood glucose) between the two groups are shown in Fig. S1.

## Results

### Characteristics of study participants

The clinical characteristics of participants were listed in Table 1. There was no statistical difference in maternal age, gestational age, gravidity, systolic blood pressure (sBP), diastolic blood pressure (dBP), parity, educational level, body mass index (BMI), family income, and delivery modes between the two groups, whilst fasting blood glucose, 60/120 minutes postprandial blood glucose after 75 g oral glucose differed significantly. Newborn characteristics, including birth weight and birth length were not significantly different between groups.

**Table 1:** Characteristics of study participants

	Controls (*n* = 20)	GDM (*n* = 20)	*P* value
**Age (years)**	36(35,37.5)	36(35.25,37)	0.933^a^
**Total years of schooling (years)**	16(15,16)	15.5(12,16)	0.343^a^
**Tertiary Education**			
Unaccepted	9(45.00)	10(50.00)	0.752^c^
Graduated	11(55.00)	10(50.00)	
**House income**			
less than 4000 RMB per month	1(5.00)	1(5.00)	
less than 7000 RMB per month	4(20.00)	4(20.00)	
less than 10000 RMB per month	7(35.00)	6(30.00)	0.492^c^
less than 16000 RMB per month	7(35.00)	5(25.00)	
less than 25000 RMB per month	0(0.00)	3(15.00)	
less than 70000 RMB per month	0(0.00)	1(5.00)	
More than 70000 RMB per month	1(5.00)	0(0.00)	
**Gravidity**	3(1.25,5)	4(3,4.75)	0.498^a^
**Parity**			
Nulliparous	8(40.00)	5(25.00)	0.32^c^
Primiparous	11(55.00)	15(75.00)	
Multiparous	1(5.00)	0(0.00)	
**BMI (kg/m**^**2**^**)**	22.08±3.6	23±2.94	0.382^b^
**sBP, mmHg**	111.8±11.11	112±8.77	0.95^b^
**dBP, mmHg**	69.65±8.02	69.5±8.16	0.954^b^
**Fasting blood glucose (mmol/L)**	4.55(4.43,4.8)	5.2(4.7,5.48)	0.001^a^
**60-min postprandial blood glucose (mmol/L)**	7.1(6.7,8.5)	10(8.38,10.90)	<0.001^a^
**120-min postprandial blood glucose (mmol/L)**	6.74±0.84	9.02±1.28	<0.001^b^
**Mode of delivery**			
Unassisted vaginal	5(25.00)	7(35.00)	0.478^c^
Operative vaginal	1(5.00)	0(0.00)	
Prelabour LSCS	0(0.00)	1(5.00)	
LSCS in labour	14(70.00)	12(60.00)	
**Delivery gestational age (weeks)**	39(38,39.75)	38(37,39)	0.075^a^
**Birth weight (g)**	3365(3172.5,3537.5)	3200(2850.5,3617)	0.304^a^
**Birth length (cm)**	50(49,50)	49(48.25,51)	0.484^a^

*Abbreviations*: *sBP* systolic blood pressure, *dBP* diastolic blood pressure, *LSCS* lower segment cesarean section

Values are means ± SD, median (IQR) or n (%)

^a^ *P* value from Mann–Whitney test

^b^ *P* value from Student t-test

^c^ *P* value from Chi square test

### Metabolite profiling of plasma and urine

**Fig. 1:**
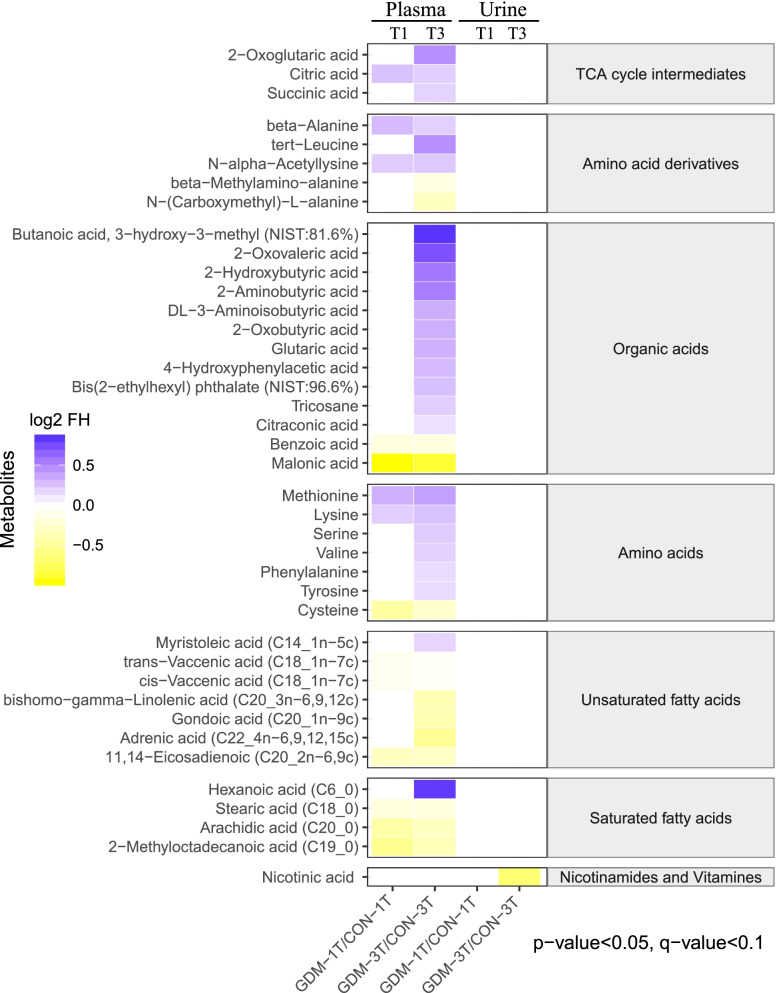
Heatmap showing the metabolome profiles of plasma and urine in the first and third trimester. The relative abundance of metabolites is illustrated on a log2 scale. Fold difference of metabolite concentrations compared with their corresponding controls are plotted as shades of purple (increasing levels) or yellow (decreasing levels). Only metabolites with a significant *p*-value (Tukey’s HSD: *p* < 0.05), q-value (FDR: q < 0.1) are shown

In total, 192 and 165 metabolites were identified in maternal urine and plasma samples respectively. Of samples in the first trimester, no differences between groups were seen in urine, while 14 metabolites were observed to be significantly different in GDM plasma samples. This included 5 metabolites (lysine, N-alpha-acetyllysine, citric acid, beta-alanine, methionine) with higher levels, while 9 with lower levels in GDM pregnancies relative to healthy controls (Fig. 1).

In the third trimester samples, one and 39 metabolites were significantly different between case and control groups in urine and plasma respectively (Fig. 1, Table S2). In urine, nicotinic acid was the only metabolite significantly lower in GDM group. In plasma, 25 metabolites were higher in GDM pregnancies, including the majority of amino acids, amino acid derivatives, and tricarboxylic acid (TCA) cycle intermediates, hexanoic acid and myristoleic acid, while 14 were lower in the case group. This included three saturated fatty acids (2-methyloctadecanoic acid, arachidic acid, stearic acid), six unsaturated fatty acids (adrenic acid, gondoic acid, bishomo-gamma-linolenic acid, 11,14-eicosadienoic acid, cis-vaccenic acid, trans-vaccenic acid), three amino acids derivatives (cysteine, N-(Carboxymethyl)-L-alanine, beta-methylamino-alanine) and two organic acids (malonic acid, benzoic acid). The greatest fold differences between the two groups in the first trimester were higher methionine and beta-alanine, and lower cysteine and arachidic acid in GDM pregnancies.

### Receiver operating characteristic curve (ROC) analysis for plasma and urine

**Fig. 2:**
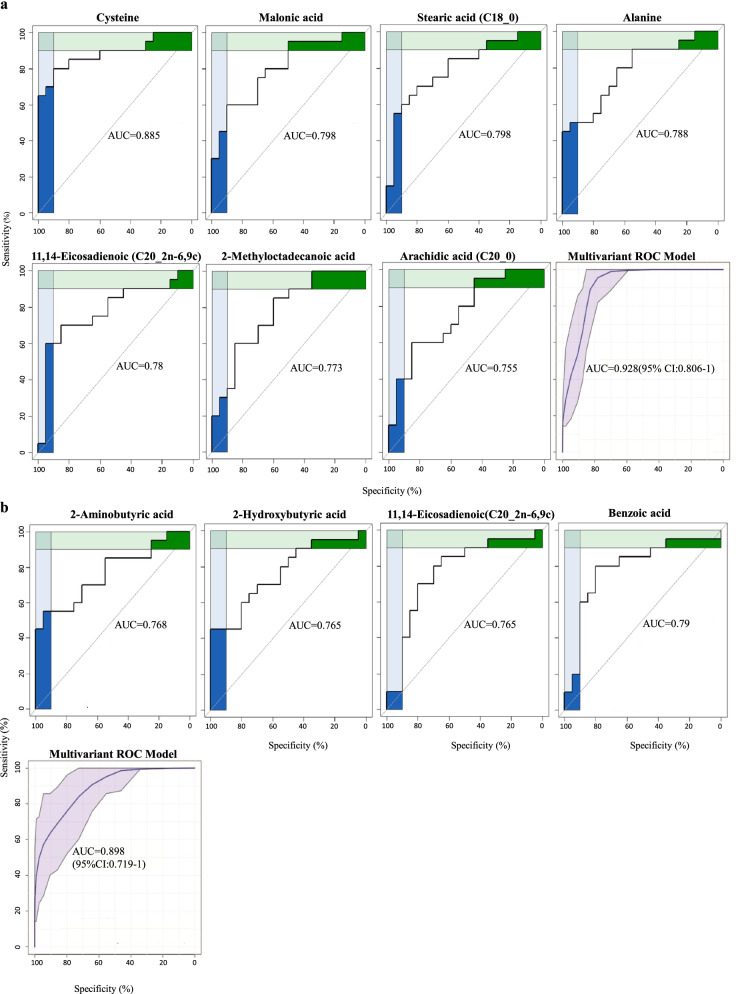
ROC curve of plasma metabolites with AUC above 0.75 between GDM and normal pregnancies. Seven metabolites in the first (**a**) and four metabolites in the third (**b**) trimester. A multivariant ROC model and corresponding 95% Confidence Interval (CI) combining the seven and four metabolites are shown in the last plot

**Fig. 3:**
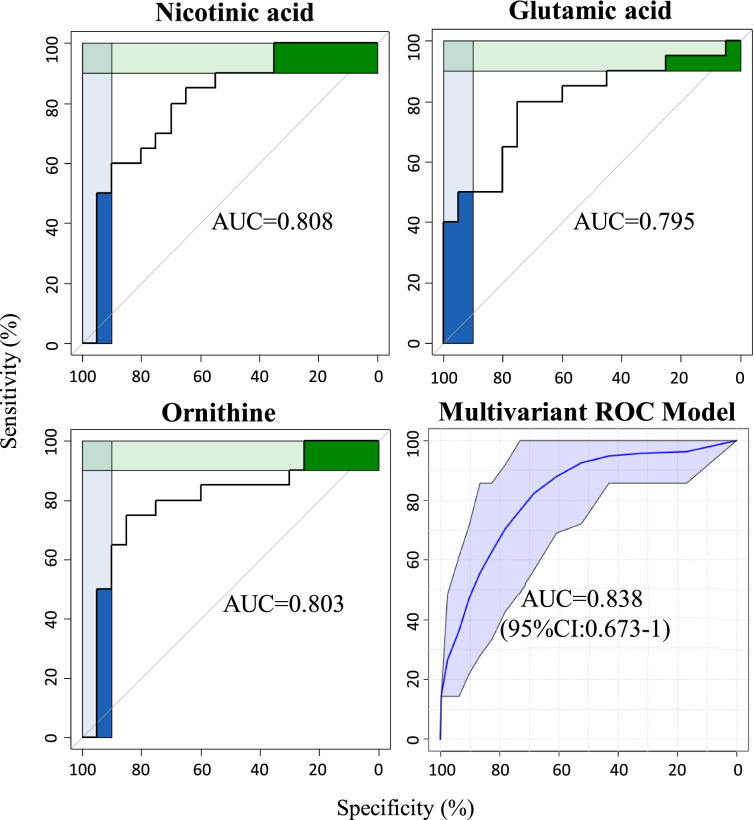
ROC curve of urine metabolites with AUC above 0.75 between GDM and normal pregnancies. Three metabolites in the third trimester between the two groups. A multivariant ROC model and corresponding 95% CI combining all of the three metabolites are plotted

With regard to plasma ROC analysis, seven and four metabolites were shortlisted in the first and third trimester respectively with an area under curve (AUC) above 0.75. These included four fatty acids (arachidic acid, stearic acid, 2-methyloctadecanoic acid, and 11,14-eicosadienoic acid), two amino acid (alanine and cysteine), and one organic acid (malonic acid) in the first trimester (Fig. 2a). Meanwhile, three organic acids (2-aminobutyric acid, 2-hydroxybutyric acid, and benzoic acid) and one unsaturated fatty acid (11,14-Eicosadienoic acid) were found in the third trimester (Fig. 2b). By combining seven and four shortlisted metabolites for the first and third trimester via multivariant ROC models, we were capable of discriminating GDM from healthy pregnancies with an AUC of 0.928 and 0.898 respectively. In terms of urine profiles, only three significant metabolites (nicotinic acid, glutamic acid, and ornithine) were identified with an AUC above 0.75 in the third trimester (Fig. 3). A multivariant ROC model combining these three metabolites was established to differentiate GDM from healthy pregnancies with an AUC of 0.838. Besides, longitudinal metabolite profiles of plasma and urine between first and third trimesters were illustrated (Fig. S2, Table S4).

### Metabolic pathway enrichment analysis

**Fig. 4:**
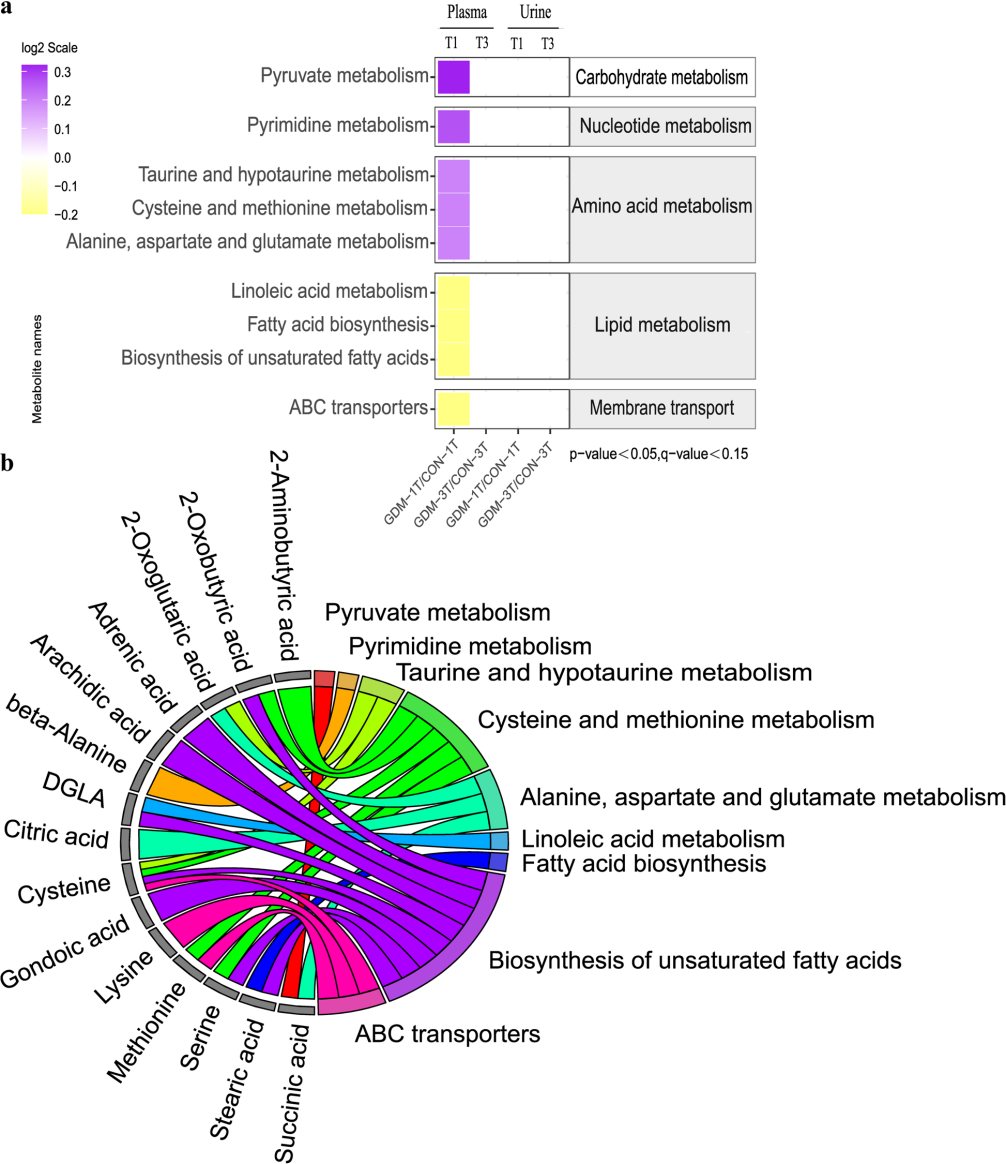
KEGG pathway analysis. The predicated pathway activities were illustrated with log2 scale (**a**). The activities of the pathways are plotted by purple color (upregulation) and yellow color (downregulation). Only metabolites with a significant *p*-value (Tukey’s HSD: *p* < 0.05), q-value (FDR: q < 0.15) are shown. The different metabolites involved in the corresponding pathways (**b**)

The identified metabolites were performed enrichment analysis using the Kyoto Encyclopedia of Genes and Genomes (KEGG) database to further characterize into functional pathway categories. The predicted outcome demonstrated that only nine metabolic pathways appeared to be significantly altered between GDM and control groups in the first trimester (Fig. 4a). The metabolism of carbohydrate, nucleotide, and amino acid were upregulated, whilst all metabolic pathways classified into lipid metabolism were downregulated in GDM group, besides, the ATP-binding cassette (ABC) transporters pathway shown the similar trend with lipid metabolism. Especially, metabolism of amino acid and fatty acid, as well as membrane transport mainly ABC transporters involving methionine, lysine and cysteine might play an important role in the pathogenesis of GDM (Fig. 4b).

## Discussion

In our study, we have chosen to discuss GDM with maternal age between 35-40 years, which is becoming one of the major sources of adverse maternal outcomes in China. MS-based metabolomics has been applied in diverse investigations of pregnancy-related complications, it is a reliable alternative to conventional biochemical assays, but has not been widely accepted. We implicated this technology to elucidate the pathophysiology of GDM in pregnant women more than 35 years, by conducting an untargeted metabolomics utilizing urine and plasma to characterize metabolic changes prior (11 to 14 gestational weeks) and post (32 to 34 gestational weeks) to the onset of GDM. We revealed several distinct plasma metabolic signatures in the first trimester that predicted subsequent GDM with the predictive power of AUC 0.928 (95%CI 0.806-1, p<0.05). The plasma metabolome displayed much more prominent metabolic changes than urine samples.

Our study indicates that plasma is a more promising biofluid than urine to discriminate GDM from normal pregnancies in women with advanced maternal age, especially in the third trimester. Cross-sectional analyses revealed that 39 plasma metabolites were significantly fluctuated throughout the GDM pregnancy accordingly, while merely one metabolite was significantly altered in urine samples (Fig. 1). This is in line with other studies related to GDM. Lorenzo-Almoros *et al* suggested that plasma sample can be implicated as predictive and diagnostic biomarkers for GDM because the plasma can provide extensive information on dysfunctional adipose tissue and placental-derived factors, which may contribute to low-grade inflammation and insulin resistance [19]. On the contrary, there are limited metabolomic biomarkers from urine to predict GDM and associate with metabolic diseases [19, 20]. This may be owing to the different metabolic nature between plasma and urine as all organs and tissues are bathed in blood, and it can represent as a reasonable metabolic proxy for an organism, which is defined as “metabolic fingerprinting” [21]. Thus, the blood sample is more ideal for studying cellular physiology and cellular processes. On the other hand, urine is the product of blood filtered by the glomerulus and reabsorbed by the renal tubules. It is considered as “metabolic footprinting”, an excreted biofluid that mainly reflects important physiology of kidney function [22]. Since all recruited participants had no kidney dysfunction in our study, little difference between GDM and healthy controls was indeed observed.

Metabolic profiling unveiled that dysregulated ABC transporters and abnormal amino acid compositions may be associated with the development of GDM among AMA women. Firstly, KEGG pathway enrichment analysis displayed GDM related amino acids, including methionine, lysine, and cysteine, were participated in the ABC transporters (Fig. 4b). A human study on population with advanced maternal age indicated that there was an inability of essential amino acid absorption by skeletal muscle after resistance exercise due to lack of expression on ABC transporters in older adults [23]. Additionally, Samantha *et al* revealed that compromised amino acid transportation in placentae was observed in AMA mice [24, 25]. Secondly, our findings on the elevated plasma methionine and decreased cysteine in GDM group may shed light on specific dietary amino acid composition for pregnancies with advanced maternal age. Previous aged animal study revealed methionine restriction and cysteine supplementation diet could ameliorate insulin resistance [26, 27]. Virginia L *et al* indicated methionine restriction could reduce insulin and homeostasis model assessment in leptin-deficient obese mice fed with methionine restriction diet intervention between 10-24 weeks of age [28]. Blouet *et al* demonstrated insulin resistant rats induced by high sucrose diet could be rescued by cysteine supplementation [29]. Interestingly, literatures suggested that the therapeutic intervention of methionine restriction and cysteine supplementation are attributable to their antioxidant capability against oxidative stress during aging. Rats fed with methionine restriction diet showed a reduction in reactive oxygen species (ROS) generation and protected mitochondrial DNA from ROS damage in liver, brain, heart, and kidney [30]. Cysteine supplementation could ameliorate insulin resistance and age-related degenerations by improving skeletal muscle functions and reducing oxidative stress [27, 29]. These outcomes highlight the importance of clinical consideration of specific nutrient composition for diet instructions to GDM women more than 35 years old.

Fatty acids are reported to negatively associate with insulin resistance [31]. In line with previous results, a reduction of fatty acid was found in plasma of elder GDM women in our study. These changes were persisted throughout pregnancy and could likely to be associated with onset of GDM. The concentrations of saturated fatty acids, including stearic acid, arachidic acid, bishomo-gamma-linolenic acid (dihomo-gamma-linolenic acid, DGLA), and adrenic acid, in maternal plasma were lower than those in control participants in the third trimester (Fig. 1). Furthermore, disturbances of lipid metabolism have been associated with inflammation occurring in GDM population with advanced maternal age. On one hand, as early in 1966, a total downregulation of fatty acid biosynthesis was found in the adipose tissue of 23 months old rats [32]. On the other hand, Marseille-Tremblay *et al* have demonstrated a lower concentration of fatty acids in placenta accompanied by elevated inflammatory markers such as IL-1β and TNF-α in GDM [23]. However, the specific signaling pathway that may link lipid metabolism with glucose homeostasis in GDM remains unclear. Future work is warranted to elucidate the exact metabolic mechanism for age-related aberrant lipid metabolism.

There were some limitations worth noting in our study. Firstly, as some samples in mid-gestation from the recruited participants are absent, we just assessed maternal metabolites at 11–14 and 32–34 gestational weeks, which precludes analysis of shifts in metabolite levels across pregnancy. Secondly, the recruited pregnant women from CLIMB were just 35-40 years old, but maternal age over 40 years is often considered of an advanced maternal age [33], future investigations involving women with more advanced maternal age (≥40 years) are warranted to verify the metabolic dysregulation of GDM with advanced maternal age. Thirdly, to validate our findings, further research is required and that valuable additional information would be yielded through the inclusion of younger women with/without GDM in the analysis. Lastly, the influence of various dietary patterns on the plasma metabolome of women with GDM should also be addressed in the future study.

## Conclusions

In conclusion, we observed that GDM women between 35-40 years have a subclinical dysmetabolism in early gestation. The potential mechanism may include defects in the metabolism of amino acid and fatty acid, and further worsening insulin resistance. We found that combination of several long-chain fatty acid and amino acids could predict GDM with AMA in the first trimester. Confirming these metabolite markers is essential for further application of MS-based metabolomics in the clinical field to accept and adopt the results from biomarker discovery studies. Additionally, the metabolomic profiling provides hint for clinical obstetricians to instruct GDM women over 35 years old for specific diet nutrient composition. Those changes in amino acid and lipid metabolism from the early gestational period are unlikely the consequence of GDM development but may be associated with onset and progression of the disease.

## Supplementary Information

**Additional file 1: Table S1.** The OR, AUC and statistical tests of maternal plasma metabolites between GDM and normal pregnancies in the first trimester. **Table S2.** The OR, AUC and statistical tests of maternal plasma metabolites between GDM and normal pregnancies in the third trimester. **Table S3.** The AUC, OR and statistical tests of maternal urine metabolites between GDM and normal pregnancies in the third trimester. **Table S4.** The relative abundance of significant plasma metabolites in the first and third trimesters between GDM and normal pregnancies (repeated measurement ANOVA). **Figure S1.** Power analysis of 75 g OGTT (120 min postprandial blood glucose) between GDM and normal pregnancy at 24–28 gestational weeks. **Figure S2.** The relative abundance of significant plasma (a-c) and urine (d) metabolites in the first and third trimesters collected from GDM and normal pregnancies.

## Data Availability

The datasets generated during this study are available from the corresponding author on reasonable request.

## Abbreviations

GDM: Gestational diabetes mellitus
AMA: Advanced maternal age
CLIMB: Complex Lipids in Mothers and Babies
GC–MS: Gas chromatography-mass spectrometry
ROC: Receiver operating characteristic curve
AUC: Area under curve
LC-MS: Liquid chromatography-mass spectrometry
BMI: Body mass index
OGTT: Oral glucose tolerance test
IADPSG: International Association of Diabetic Pregnancy Study Group
QC: Quality controls
MS: Mass spectrometry
IS: Internal standard
FDR: False discovery rates
CI: Confidence interval
KEGG: Kyoto Encyclopedia of Genes and Genomes
TCA cycle: Tricarboxylic acid cycle
ABC transporters: ATP-binding cassette transporters
ROS: Reactive oxygen species
DGLA: Bishomo-gamma-linolenic acid (dihomo-gamma-linolenic acid)
ANOVA: Analysis of variance
