# Supplementary material for: The effects of gestational diabetes mellitus with maternal age between 35 and 40 years on the metabolite profiles of plasma and urine
Source: BMC Pregnancy Childbirth. 2022 Mar 2;22:174. doi: 10.1186/s12884-022-04416-5 (PMC8892719; doi:10.1186/s12884-022-04416-5)
Supplement: Supplementary file 1 — Additional file 1: Table S1. The OR, AUC and statistical tests of maternal plasma metabolites between GDM and normal pregnancies in the first trimester. Table S2. The OR, AUC and statistical tests of maternal plasma metabolites between GDM and normal pregnancies in the third trimester. Table S3. The AUC, OR and statistical tests of maternal urine metabolites between GDM and normal pregnancies in the third trimester. Table S4. The relative abundance of significant plasma metabolites in the first and third trimesters between GDM and normal pregnancies (repeated measurement ANOVA). Figure S1. Power analysis of 75 g OGTT (120 min postprandial blood glucose) between GDM and normal pregnancy at 24–28 gestational weeks. Figure S2. The relative abundance of significant plasma (a-c) and urine (d) metabolites in the first and third trimesters collected from GDM and normal pregnancies. [file 12884_2022_4416_MOESM1_ESM.pdf]

Table S1. The OR, AUC and statistical tests of maternal plasma metabolites between GDM and normal pregnancies in the first trimester

Metabolites	OR	CI_lower	CI_upper	AUC	*P* value	qvalue
11,14-Eicosadienoic	0.9998	0.9997	0.9999	0.78	0.0038	0.0577
2-Methyloctadecanoic acid	0.9989	0.9981	0.9996	0.7725	0.0008	0.0288
Arachidic acid	0.9995	0.9991	0.9998	0.755	0.0046	0.0577
Benzoic acid	0.9996	0.9992	0.9999	0.7125	0.0144	0.0649
beta-Alanine	1.0006	1.0000	1.0013	0.7225	0.0186	0.0699
cis-Vaccenic acid	0.9999	0.9999	0.9999	0.73	0.0108	0.0577
Citric acid	1.0000	1.0000	1.0000	0.7375	0.0139	0.0649
Cysteine	0.9991	0.9985	0.9996	0.885	1.19E-05	0.0012
Lysine	1.0000	1.0000	1.0000	0.715	0.0105	0.0577
Malonic acid	0.9969	0.9942	0.9989	0.7975	0.0011	0.0288
Methionine	1.0001	1.0000	1.0003	0.7225	0.0096	0.0577
N-alpha-Acetyllysine	1.0004	1.0000	1.0009	0.6825	0.0138	0.0649
Stearic acid	0.9999	0.9999	0.9999	0.7975	0.0014	0.0296
trans-Vaccenic acid	0.9999	0.9999	0.9999	0.73	0.0108	0.0577

Abbreviation: OR, odds ratios; AUC, area under the curve; CI, confidence intervals.

Table S2. The OR, AUC and statistical tests of maternal plasma metabolites between GDM and normal pregnancies in the third trimester

Metabolites	OR	CI_lower	CI_upper	AUC	*P* value	qvalue
Benzoic acid	0.9994	0.9989	0.9998	0.79	0.0023	0.0296
2-Aminobutyric acid	1.0000	1.0000	1.0000	0.7675	0.0023	0.0296
2-Hydroxybutyric acid	1.0000	1.0000	1.0001	0.765	0.0029	0.0296
Methionine	1.0001	1.0000	1.0002	0.7425	0.0051	0.0420
Cysteine	0.9996	0.9992	0.9999	0.72	0.0087	0.043
Arachidic acid	0.9994	0.9989	0.9998	0.735	0.0098	0.043
2-Oxoglutaric acid	1.0004	1.0001	1.0009	0.7125	0.0134	0.043
tert-Leucine	1.0004	1.0001	1.0009	0.7125	0.0134	0.043
11,14-Eicosadienoic	0.9999	0.9998	0.9999	0.765	0.0145	0.043
Stearic acid	0.9999	0.9999	1	0.7275	0.0150	0.043
Adrenic acid	0.9998	0.9997	0.9999	0.7325	0.0158	0.043
Lysine	1.0000	1.0000	1.0000	0.705	0.0183	0.0465
Valine	1.0000	1	1.0000	0.695	0.0194	0.0465
N-alpha-Acetyllysine	1.0004	1.0000	1.001	0.6475	0.0245	0.0529
Myristoleic acid	1.0016	1.0001	1.0035	0.695	0.0247	0.0529
2-Methyloctadecanoic acid	0.9995	0.9990	1.0000	0.715	0.0267	0.0542
Tricosane	1.0005	1.0000	1.0011	0.7	0.0304	0.0568
Citric acid	1.0000	1.0000	1.0000	0.705	0.0316	0.0568
Glutaric acid	1.0001	1.0000	1.0004	0.6475	0.0333	0.0568
Aminoisobutyric acid	1.0001	1.0000	1.0002	0.655	0.0355	0.0568

Malonic acid	0.9991	0.9976	1.0000	0.705	0.0377	0.0568
2-Oxobutyric acid	1.0004	0.9999	1.0011	0.695	0.0381	0.0584
DGLA	0.9999	0.9999	1	0.7175	0.0386	0.0650
beta-Alanine	1.0008	0.9999	1.0018	0.6575	0.0387	0.0693
Citraconic acid	1.0010	0.9999	1.0023	0.6675	0.0400	0.0693
Hexanoic acid	1.0000	1.0000	1.0000	0.7	0.0407	0.0693
Phenylalanine	1.0000	0.9999	1.0000	0.6625	0.0410	0.0699
Gondoic acid	0.9999	0.9999	1	0.71	0.0422	0.0729
Serine	1.0008	0.9999	1.0020	0.65	0.0423	0.0729
Succinic acid	1.0001	0.9999	1.0003	0.65	0.0426	0.0818
N-(Carboxymethyl)-L-alanine	0.9978	0.9947	1.0004	0.65	0.0427	0.0867
Tyrosine	1.0000	0.9999	1.0000	0.6575	0.0429	0.0867
2-Oxovaleric acid	1.0000	1.0000	1.0000	0.69	0.0434	0.0940
beta-Methylamino-alanine (BMAA)	0.9989	0.9968	1.0007	0.69	0.0435	0.0964
4-Hydroxyphenylacetic acid	1.0004	0.9997	1.0012	0.63	0.0448	0.0993
cis-Vaccenic acid	0.9999	0.9999	1.0000	0.6425	0.0446	0.0993
trans-Vaccenic acid	0.9999	0.9999	1.0000	0.6425	0.0496	0.0993
Butanoic acid	1.0002	0.9979	1.0002	0.65	0.0497	0.0995
Bis(2-ethylhexyl) phthalate	1.0001	0.9999	1.0003	0.71	0.0499	0.0997

Abbreviation: DGLA, dihomo-gamma-linolenic acid

Table S3. The AUC, OR and statistical tests of maternal urine metabolites between GDM and normal pregnancies in the third trimester

Metabolites	OR	CI_lower	CI_upper	AUC	*P* value	qvalue
Nicotinic acid	0.9961	0.9929	0.9985	0.8075	0.0005	0.0722
Ornithine	0.9999	0.9999	0.9999	0.8025	0.0442	0.4491
Glutamic acid	0.9998	0.9998	0.9999	0.795	0.0078	0.4491

Table S4. The relative abundance of significant plasma metabolites in the first and third trimesters between GDM and normal pregnancies (repeated measurement ANOVA)

Metabolites	Between subject GDM/Normal	Between subject time	Within subject time	Within subject outcome: time
2-Oxobutyric acid	0.8435	NA	0.2419	0.0032
Cysteine	0.0001	NA	0.0001	0.0034
Citraconic.acid	0.2546	NA	0.0052	0.0126
L-alanine	0.6740	NA	0.0660	0.0201
dimethyltetradecanoic acid	0.3143	NA	0.0005	0.0217
Palmitic acid	0.3143	NA	0.0005	0.0217

Lactic.acid	0.0329	NA	0.5925	0.0266
2-Hydroxybutyric acid	0.0673	NA	0.0002	0.0270
Hippuric.acid	0.6753	NA	0.3139	0.0302
Glutamine	0.0598	NA	0.0064	0.0312
Oleic acid	0.4812	NA	0.1467	0.0374
EDTA	0.5963	NA	0.2712	0.0455
2-Aminobutyric acid	0.0130	NA	0.3855	0.0457

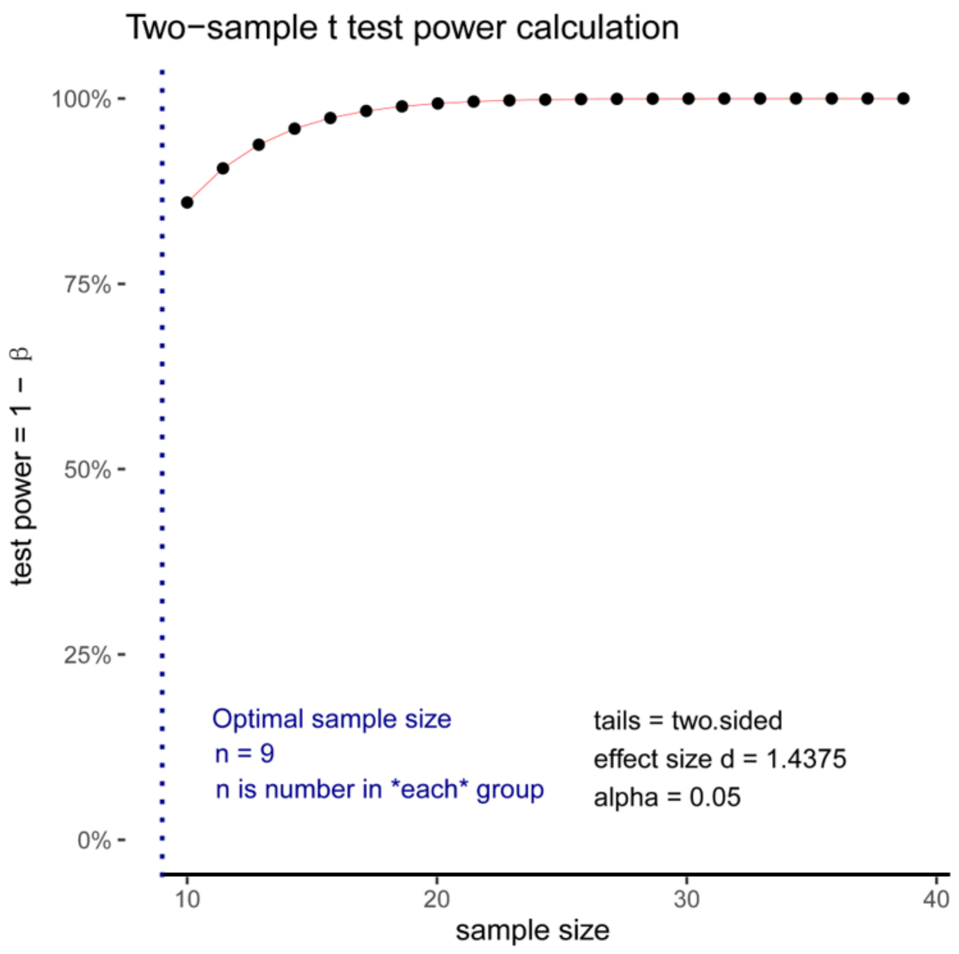

Fig. S1. Power analysis of 75g OGTT (120 min postprandial blood glucose) between GDM and normal pregnancy at 24-28 gestational weeks. The curve displays the relationship between changes of power (y-axis) along with changes in sample size (x-axis). The blue vertical line indicates 9 sample size required to have 80% power with an alpha value less than 0.05 for a given mean difference between two groups using a two-sided Student's T test. n= number. AUC = area under the curve.

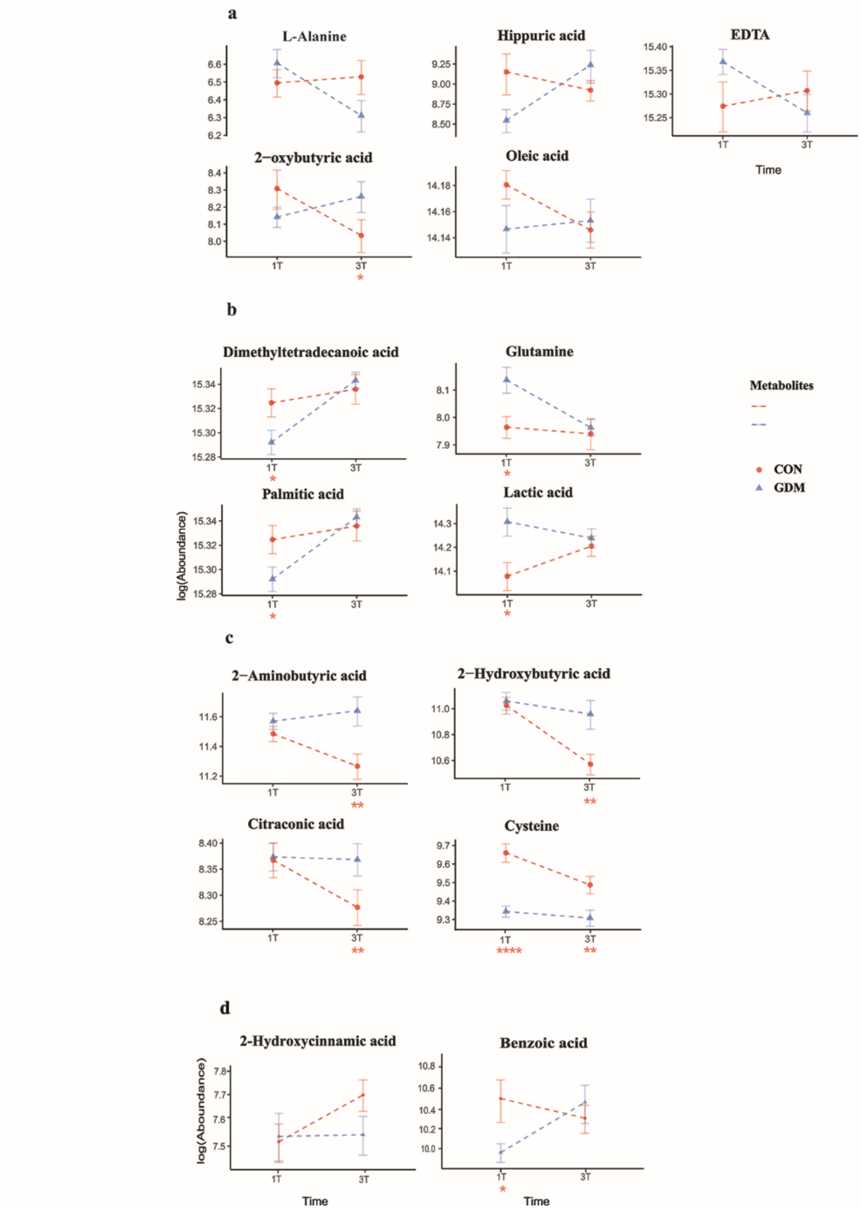

Fig. S2. The relative abundance of significant plasma (a-c) and urine (d) metabolites in the first and third trimesters collected from GDM and normal pregnancies. a) Opposite trend between first and third trimesters. b) Only displayed disparity in the first trimester. c) only discrimination in the third trimester. d) two urine metabolites shown difference between early and late trimesters. The relative abundances of metabolites were illustrated with log scale. Blue triangles represent metabolites levels collected from women with GDM. Red circles represent metabolites levels from normal pregnancies. Only the significant interactions between GDM outcomes and trimester periods are shown ($p<0.05$, repeated measurement ANOVA). Red asterisks indicate metabolites with significantly different levels in GDM and normal pregnancies with regard to the corresponding trimester. (“*”, “**”, “****” represent *P*-value less than 0.05, 0.01 and 0.0001 respectively).
